# 

**Published:** 1884-06

**Authors:** 


### CONTENTS.

ORIGINAL COMMUNICATIONS—

Phthisis Pulmonalis. By H. V. M. Miller, M. D., Atlanta, Ga. . 193
Trismus Nascentium. By A. W. Griggs, M. D., West Point, Ga. . 206
Two Remarkable Surgical Cases. By W. F. Westmoreland, M. D., Atlanta, Ga., *Illustrated* . . . . . . . . . . 212
Children and their Management in Disease. By T. J. Word, M. D., Atlanta, Ga. . . . . . . . . . . . . . 222
An Ovariotomy. By Seth N. Jordan, Columbus, Ga. . . . . . . 226

CORRESPONDENCE—

The New Journal—Its Contents, etc. By E. F. Starr, M. D., Nacoochee, Ga. . . . . . . . . . . . . . . . . 230

SOCIETY PROCEEDINGS—

American Medical Association. . . . . . . . . . . . . . . . 234

EDITORIAL—

Death of Prof. Samuel D. Gross. . . . . . . . . . . . . 249
Dr. Henry F. Campbell. . . . . . . . . . . . . . . . . . 252
Our Portrait Gallery. . . . . . . . . . . . . . . . . . . 253
Rectal Etherization. . . . . . . . . . . . . . . . . . . . 254

NOTES— . . . . . . . . . . . . . . 205, 211, 221, 225, 229, 248, 256.

